# Supplementary material for: Robotic Rectal Cancer Surgery: Perioperative and Long-Term Oncological Outcomes of a Single-Center Analysis Compared with Laparoscopic and Open Approach
Source: Cancers (Basel). 2025 Mar 2;17(5):859. doi: 10.3390/cancers17050859 (PMC11898783; doi:10.3390/cancers17050859)
Supplement: Supplementary file 1 [file cancers-17-00859-s001.zip › cancers-3438688-supplementary.pdf]

cancers

Table S1. Subgroup analysis for mid and low rectal cancer patients with neoadjuvant radiation.

Variable	All cohort (n = 197)	LAP (n = 82)	Open (n = 57)	Robot (n = 58)	*p*-value
Clinical stage					0.235
Stage 1	5 (2.5%)	3 (3.7%)	2 (3.5%)	0 (0%)	
Stage 2	43 (21.8%)	14 (17.1%)	17 (29.8%)	12 (20.7%)	
Stage 3	149 (75.6%)	65 (79.3%)	38 (66.7%)	46 (79.3%)	
Procedure					**0.001**
Abdomino-perineal resection	31 (15.7%)	10 (12.2%)	3 (5.3%)	18 (31%)	
Anterior resection	5 (2.5%)	2 (2.4%)	3 (5.3%)	0 (0%)	
Low anterior resection (TME)	161 (81.7%)	70 (85.4%)	51 (89.5%)	40 (69%)	
Stoma created	194 (98.5%)	81(98.8%)	56(98.2%)	57 (98.3%)	0.209
Additional procedure	34 (17.3%)	13 (15.9%)	18 (31.6%)	3 (5.2%)	**0.001**
BSO	17 (8.6%)	5 (6.1%)	11 (19.3%)	1 (1.7%)	**0.002**
Type of anastomosis					**<0.001**
Colonic pouch	9 (5.8%)	3 (4.5%)	5 (10.2%)	1 (2.5%)	
End-to-end	109 (69.9%)	52 (77.6%)	21 (42.9%)	36 (90%)	
Hand-sewn coloanal	18 (11.5%)	7 (10.4%)	8 (16.3%)	3 (7.5%)	
Side-to-end	20 (12.8%)	5 (7.5%)	15 (30.6%)	0 (0%)	
Skin incision					**0.023**
Left lower quadrant	2 (1.4%)	2 (2.4%)	-	0 (0%)	
Midline laparotomy	22 (15.7%)	18 (22%)	-	4 (6.9%)	
Natural orifice	41 (29.3%)	22 (26.8%)	-	19 (32.8%)	
Periumbilical	3 (2.1%)	3 (3.7%)	-	0 (0%)	
Pfanensteil	72 (51.4%)	37 (45.1%)	-	35 (60.3%)	
Conversion	23 (16.4%)	19 (23.2%)	-	4 (6.9%)	**0.011**
Intraoperative complication	37 (18.8%)	13 (15.9%)	17 (29.8%)	7 (12.1%)	**0.033**
Bleeding	9 (4.6%)	2 (2.4%)	7 (12.3%)	0 (0%)	**0.004**
LOS, days, median (range)	8 (4–73)	8 (4–73)	10 (6–47)	7 (5–53)	**<0.001**
Postoperative complications, overall	144 (73.1%)	57 (69.5%)	48 (84.2%)	39 (67.2%)	0.085
SSI	28 (14.2%)	8 (9.8%)	15 (26.3%)	5 (8.6%)	**0.008**
Ileus/SBO	46 (23.4%)	18 (22%)	19 (33.3%)	9 (15.5%)	0.075

Variable	All cohort (n = 197)	LAP (n = 82)	Open (n = 57)	Robot (n = 58)	*p*-value
Electrolyte disturbances/ARF	84 (42.6%)	34 (41.5%)	22 (38.6%)	28 (48.3%)	0.576
Clavien–Dindo score, median (range)	1 (0–5)	1 (0–4)	2 (0–5)	1 (0–4)	**0.013**
Major complications (Clavien–Dindo > 2)	41 (20.8%)	14 (17.1%)	17 (29.8%)	10 (17.2%)	0.144
Distal margin, cm, median (range)	2 (0.2–9)	2.1 (0.2–9)	1.5 (0.3–5.5)	2.7 (0.3–6)	0.155
Disease recurrence	51 (25.9%)	20 (24.4%)	14 (24.6%)	20 (29.3%)	0.625
Local recurrence	11 (5.6%)	3 (3.7%)	5 (8.8%)	3 (5.2%)	
Distant recurrence	40 (20.3%)	17 (20.7%)	9 (15.8%)	14 (24.1%)	
Overall mortality during follow-up time	19 (9.6%)	8 (9.8%)	7 (12.3%)	4 (6.9%)	0.627

Table S2. Subgroup analysis for upper rectal cancer patients.

Variable	All cohort (n = 297)	LAP (n = 186)	Open (n = 71)	Robot (n = 40)	*p*-value
Clinical stage					0.257
Stage 1	96 (37.5%)	70 (42.7%)	17 (29.3%)	9 (26.5%)	
Stage 2	59 (23%)	35 (21.3%)	15 (25.9%)	9 (26.5%)	
Stage 3	101 (39.5%)	59 (36%)	26 (44.8%)	16 (47.1%)	
Procedure					0.41
Anterior resection	167 (56.4%)	109 (58.9%)	39 (54.9%)	19 (47.5%)	
Low anterior resection (TME)	129 (43.6%)	76 (41.1%)	32 (45.1%)	21 (52.5%)	
Stoma created	101 (34%)	57(30.5%)	27(38%)	17 (42.5%)	0.585
Additional procedure	40 (13.5%)	14 (7.5%)	22 (31%)	4 (10%)	**<0.001**
BSO	19 (6.4%)	5 (2.7%)	13 (18.3%)	1 (2.5%)	<0.001
Type of anastomosis					**<0.001**
Colonic pouch	3 (1%)	1 (0.6%)	0 (0%)	2 (5.1%)	
End-to-end	234 (81%)	155 (85.6%)	44 (63.8%)	35 (89.7%)	
Hand-sewn coloanal	5 (1.7%)	4 (2.2%)	1 (1.4%)	0 (0%)	
Side-to-end	47 (16.3%)	21 (11.6%)	24 (34.8%)	2 (5.1%)	
Skin incision					0.069
Left lower quadrant	5 (2.2%)	5 (2.7%)	-	0 (0%)	
Midline laparotomy	42 (18.6%)	40 (21.5%)	-	2 (5%)	

Variable	All cohort (n = 297)	LAP (n = 186)	Open (n = 71)	Robot (n = 40)	*p*-value
Natural orifice	4 (1.8%)	4 (2.2%)	-	0 (0%)	
Periumbilical	17 (7.5%)	13 (7%)	-	4 (10%)	
Pfanensteil	158 (69.9%)	124 (66.7%)	-	34 (85%)	
Conversion	45 (19.9%)	42 (22.6%)	-	3 (7.5%)	**0.046**
Intraoperative complication	37 (12.5%)	22 (11.8%)	12 (16.9%)	3 (7.5%)	0.358
Bleeding	11 (3.7%)	4 (2.2%)	5 (7%)	2 (5%)	0.164
LOS, days, median (range)	8 (3–86)	8 (3–86)	9 (4–63)	7 (4–16)	**<0.001**
Postoperative complications, overall	179 (60.3%)	101 (54.3%)	51 (71.8%)	27 (67.5%)	**0.025**
SSI	41 (13.8%)	21 (11.3%)	18 (25.4%)	2 (5%)	**0.003**
Ileus/SBO	61 (20.5%)	34 (18.3%)	21 (29.6%)	6 (15%)	0.093
Electrolyte disturbances/ARF	89 (30%)	43 (23.1%)	31 (43.7%)	15 (37.5%)	**0.004**
Clavien–Dindo score, median (range)	1 (0–5)	1 (0–5)	1 (0–5)	1 (0–3)	**0.045**
Major complications (Clavien–Dindo > 2)	35 (11.8%)	21 (11.3%)	12 (16.9%)	2 (5%)	0.169
Distal margin, cm, median (range)	3 (0.2–7.5)	3 (0.6–7.5)	2 (0.2–5)	4.5 (2.5–7)	**0.002**
Disease recurrence	70 (23.6%)	39 (21%)	20 (28.2%)	11 (27.5%)	0.655
Local recurrence	24 (8.1%)	13 (7%)	8 (11.3%)	3 (7.5%)	
Distant recurrence	46 (15.5%)	26 (14%)	12 (16.9%)	8 (20%)	
Overall mortality during follow-up time	30 (10.1%)	17 (9.1%)	9 (12.7%)	4 (10%)	0.727

Figure S1: Overall survival (A) and recurrence-free survival (B) after subgroup analysis for low and mid rectal cancer treated with neoadjuvant therapy, and upper rectal cancer.

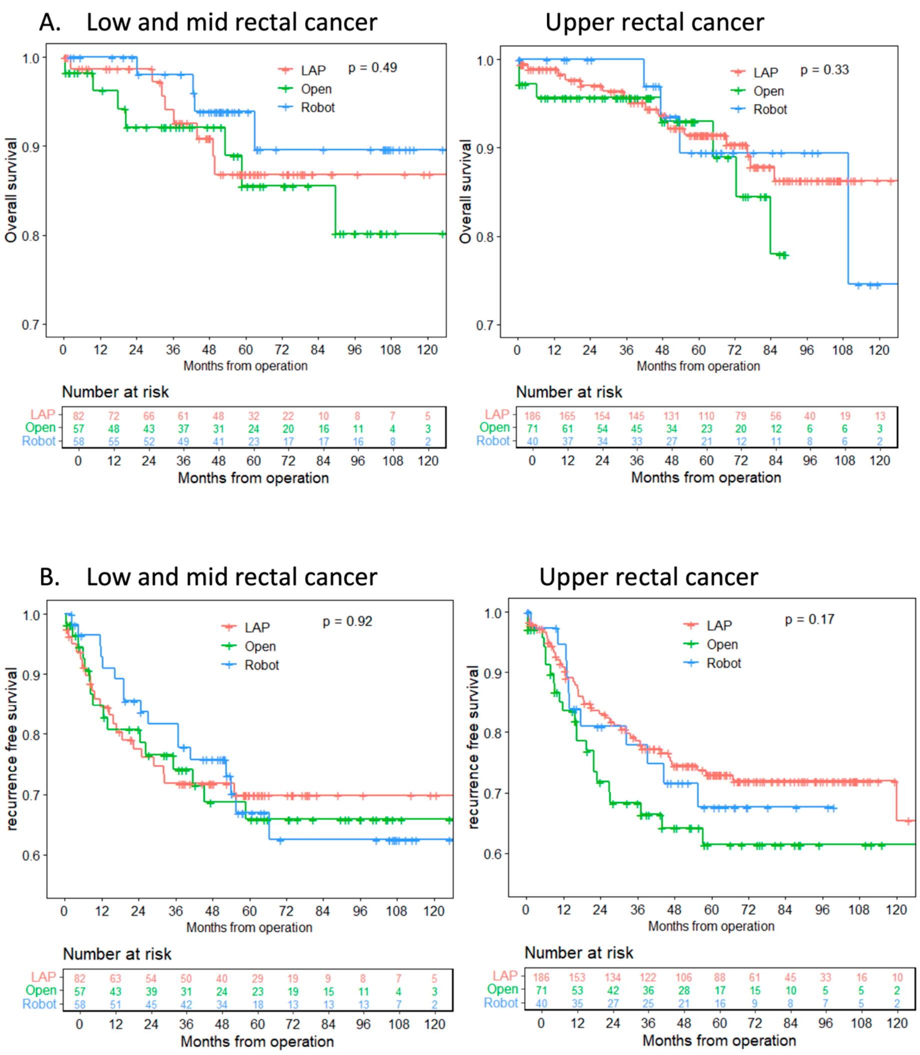
A. Low and mid rectal cancer
Upper rectal cancer
Overall survival
Months from operation
LAP
Open
Robot
p = 0.49
p = 0.33
Number at risk
LAP 82 72 66 61 48 32 22 10 8 7 5
Open 57 48 43 37 31 24 20 16 11 4 3
Robot 58 55 52 49 41 23 17 17 16 8 2
LAP 186 165 154 145 131 110 79 56 40 19 13
Open 71 61 54 45 34 23 20 12 6 6 3
Robot 40 37 34 33 27 21 12 11 8 6 2
B. Low and mid rectal cancer
Upper rectal cancer
recurrence free survival
Months from operation
p = 0.92
p = 0.17
Number at risk
LAP 82 63 54 50 40 29 19 9 8 7 5
Open 57 43 39 31 24 23 19 15 11 4 3
Robot 58 51 45 42 34 18 13 13 13 7 2
LAP 186 153 134 122 106 88 61 45 33 16 10
Open 71 53 42 36 28 17 15 10 5 5 2
Robot 40 35 27 25 21 16 9 8 7 5 2
